# Progress in 50 years of viroid research—Molecular structure, pathogenicity, and host adaptation

**DOI:** 10.2183/pjab.97.020

**Published:** 2021-07-31

**Authors:** Teruo SANO

**Affiliations:** *1 Faculty of Agriculture and Life Science, Hirosaki University, Hirosaki, Aomori, Japan.

**Keywords:** viroid, non-coding RNA, functional RNA, structural motif, pathogenicity, host adaptation

## Abstract

Viroids are non-encapsidated, single-stranded, circular RNAs consisting of 246–434 nucleotides. Despite their non-protein-encoding RNA nature, viroids replicate autonomously in host cells. To date, more than 25 diseases in more than 15 crops, including vegetables, fruit trees, and flowers, have been reported. Some are pathogenic but others replicate without eliciting disease. Viroids were shown to have one of the fundamental attributes of life to adapt to environments according to Darwinian selection, and they are likely to be living fossils that have survived from the pre-cellular RNA world. In 50 years of research since their discovery, it was revealed that viroids invade host cells, replicate in nuclei or chloroplasts, and undergo nucleotide mutation in the process of adapting to new host environments. It was also demonstrated that structural motifs in viroid RNAs exert different levels of pathogenicity by interacting with various host factors. Despite their small size, the molecular mechanism of viroid pathogenicity turned out to be more complex than first thought.

## Preface—Viroids and viroid diseases

### General features of viroid RNAs.

**Figure 1.**
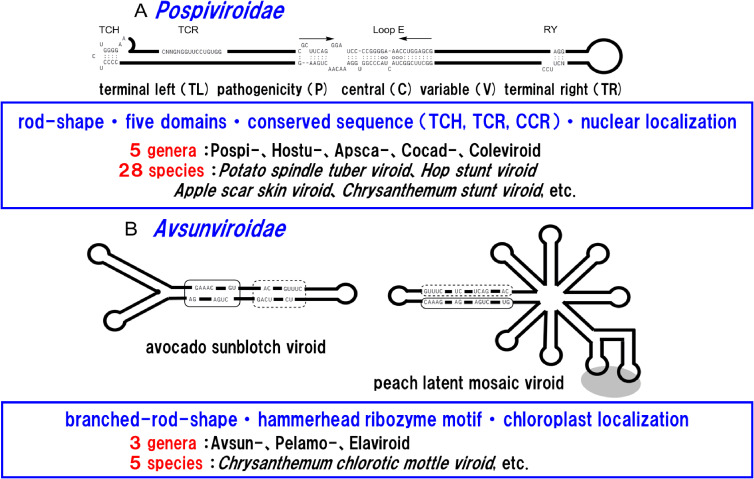
(Color online) Classification of viroids.

Viroids are tiny, non-encapsidated, single-stranded, circular RNAs. The viroid genome, consisting of 246–434 nucleotides, is a non-protein-encoding RNA; however, it autonomously replicates when it invades a host cell. Some viroids are pathogenic, but others replicate without eliciting disease symptoms. Theodor Diener, in 1971, gave the name to the causal agent of spindle tuber disease of potato (*Solanum tuberosum*). The entire nucleotide sequence was determined in 1978, and the potato spindle tuber viroid (PSTVd) was found to be a new class of pathogen consisting of a single-stranded covalently closed circular RNA genome of 359 nucleotides with high intramolecular base pairing that results in the formation of a highly structured double-stranded RNA-like rod-shaped molecule. To date, more than 30 species of viroid have been reported exclusively from angiosperms. They are classified into eight genera in two families, *Pospiviroidae* and *Avsunviroidae*, based on multiple criteria: subcellular localization [nucleus or plastids (mostly chloroplasts)]; mode of replication (symmetric or asymmetric rolling circle); the nucleotide sequence of the central conserved region; presence or absence of conserved sequences or motifs (*i.e.*, the terminal conserved sequence, the terminal conserved hairpin, or ribozyme motif); the overall identity of the nucleotide sequence (an arbitrary limit of less than 90% for species differentiation); and biological features such as host specificity. Members of *Pospiviroidae* replicate in nuclei using the asymmetrical rolling-circle method and share a central conserved region characteristic to members of the genus. In contrast, members of *Avsunviroidae* replicate in the chloroplast via a symmetrical rolling circle and have a conserved sequence of the hammerhead ribozyme. Viroids are classified as described above according to the rules for classification of viruses and are placed as subviral RNA pathogens in the viral taxonomy (Fig. 1).^1–5)^

### Impact of viroid disease.

To date, more than 25 diseases of more than 15 crops, including vegetables, fruit trees, and flowers, have been reported, including potato spindle tuber, tomato chlorotic dwarf, citrus exocortis, hop stunt, coconut cadang-cadang, apple scar skin, avocado sunblotch, and chrysanthemum stunt. The symptom development of viroid diseases is rather slow and chronic, including dwarfing and leaf malformation of plants in vegetables and ornamental flowers, abnormal enlargement and discoloration of fruit and pericarp in fruit trees, and abnormal biosynthesis of secondary metabolites. *Cadang-cadang* disease of coconut in the Philippines has killed tens of thousands of coconut trees so far.^6)^ Hop stunt disease in hops (*Humulus lupulu*s) in Japan caused great economic losses to the domestic hop industry.^7)^ In addition, potato spindle tuber and related viroids are still a threat to potato production around the world.^8,9)^

Viroids were first discovered as the smallest infectious agent of plants, with the anomalous property of having naked RNA that replicates autonomously and has one of the fundamental attributes of life to adapt to the environment according to Darwinian selection. Therefore, viroids are likely to be “living fossils” that have survived from the pre-cellular RNA world.^10)^ Although just a blink of an eye in the hundreds of millions of years of viroid evolutionary history, this paper will discuss the progress made in 50 years of viroid research, focusing on the pathogenicity and ecology of viroids elucidated mainly for the members of the *Pospiviroids*.

## 1 Structural motifs and functions for pathogenicity of viroid RNAs

Viroids, the smallest known pathogen, are unique functional non-coding RNAs that replicate autonomously in invaded host cells and incite disease in susceptible host plants. Among them, members of the genus *Pospiviroid* in the family *Pospiviroidae* are a potential risk to major crops such as potatoes and tomatoes (*Solanum lycopersicum*). Exhaustive analysis on the molecular functions related to replication and pathogenicity has been conducted, mainly using PSTVd and citrus exocortis viroid (CEVd) as models. This division outlines the advances in research on the nucleotide sequences and motifs associated with viroid functions and host interactions that have been elucidated so far.

### 1.1 Nucleotide sequence and secondary structure—Covalently closed single-stranded circular RNA.

In the early days of viroid research, accumulated data leading up to the discovery of viroids indicated that the causal agent of potato spindle tuber disease was a protein-free RNA molecule 50–80 times smaller than the smallest viral genome.^11–15)^ Theodor Diener, a discoverer of this agent, named the causal agent of potato spindle tuber disease the potato spindle tuber “viroid”.^1)^ The causal agent of CEVd, discovered independently as an infectious free nucleic acid, was also shown to be soluble in 2 M LiCl and resistant to heat, nuclease digestion, and concentrations of diethylpyrocarbonate that inactivate single-stranded RNAs, suggesting that the agent is a low molecular weight “tRNA-like” RNA with a highly ordered structure.^16,17)^ In 1973, PSTVd was first visualized using an electron microscope as a short, rod-shaped molecule in native conditions and as an open circular molecule in heat-denatured conditions.^18)^ The novel pathogen was then shown to be a covalently closed single-stranded circular RNA molecule with a molecular weight of 110,000–127,000 Da that showed high thermal stability by forming a rod-like native structure with high self-complementarity.^19)^ In 1978, the complete nucleotide sequence of PSTVd was determined by RNA fingerprint analysis, a state-of-the-art technology at that time. A secondary structure model was proposed simultaneously. PSTVd became the first eukaryotic pathogen whose entire genome had been sequenced.^20)^ The PSTVd genomic RNA is composed of 359 nucleotides that, as predicted, can form a highly structured rod-shaped stem–loop because of the high amount of intramolecular base pairing. It was also revealed that PSTVd RNA does not encode a protein, indicating that it belongs to a novel class of pathogens different from viruses. Once the nucleotide sequence was deciphered, and a secondary structure model was proposed, it became possible to analyze the function of viroids based on their nucleotide sequences and molecular structures. Severe and mild forms of PSTVd were isolated, and plants infected by mild strains were shown to be protected from developing symptoms following subsequent inoculation with severe strains, a phenomenon called cross-protection.^21)^ Comparison of severe and mild pathogenic PSTVd variants revealed that three nucleotide substitutions at different sites of the molecule might convert a pathogenic viroid to a practically non-pathogenic type.^22)^ Sequence analysis of various field isolates of PSTVd of different virulence showed that they differ by only a few nucleotides in two distinct regions of the rod-shaped molecule. Thermodynamic calculations revealed that the region located on the left-hand portion of the rod-like secondary structure of the PSTVd molecule, denoted as the virulence modulating (VM) region, becomes increasingly unstable with increasing virulence of the corresponding isolate, implying that the nucleotides of the VM region specify and modulate binding (and hence the competition potential) of the PSTVd RNA for still unknown host factors and thus determine the virulence of PSTVd.^23)^

In the meantime, molecular cloning and nucleotide sequencing of multiple CEVd isolates (A, C, DE25, DE26, DE27, DE30, and J) revealed that the length of genomic RNA of the sequence variants of CEVd varied from 370 to 375 nucleotides. Some of these isolates had nucleotide sequences that consisted of a mixture of sequence variants; they are now known as quasi-species and can be grouped into classes A and B, which differ by a minimum of 26 nucleotides primarily in two regions, named pathogenicity L (P_L_) and pathogenicity R (P_R_), of the predicted secondary structure with a total of 370–375 nucleotides. The members of classes A and B incite severe and mild disease symptoms in tomato, respectively, and P_L_ was determined to be the pathogenicity-modulating region. On the other hand, the P_R_ region was suggested to affect the efficiency of viroid infection or the replication process in plants.^24–26)^

**Figure 2.**
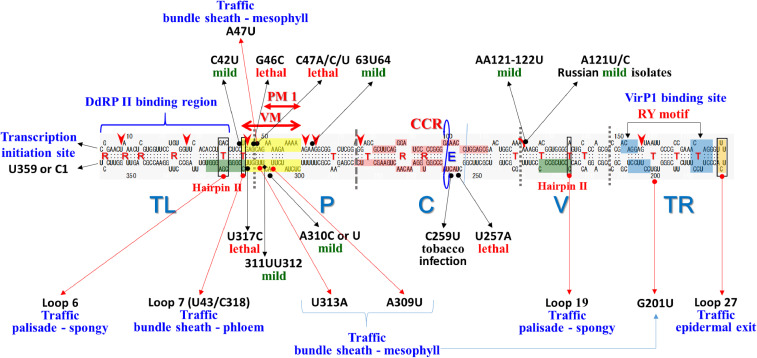
(Color online) A genetic map of PSTVd with nucleotide sequences and structural motifs regulating replication, pathogenicity, and trafficking. TL, P, C, V, and TR indicate five structural domains. T and R shown on the secondary structure indicate the loops involved in trafficking and replication, respectively. The E and blue curves in the center of the molecule indicate the position of tetraloop and loop E. Regions with green, yellow, pink, and blue backgrounds indicate the hairpin II, VM, central conserved region (CCR), and RY motifs, respectively. Nucleotide sequences with black and red arrows indicate specific sequences involved in pathogenicity and trafficking, respectively. Red arrowheads indicate G/U pairs essential for infectivity. The text in blue is a brief description of each function.

A model was proposed for five structural and functional domains in viroids based on these results, and the sequence homology of eight viroid species and more than 30 variants sequenced so far: a conserved central region (C) that may regulate the replication cycle, a region associated with pathogenicity (P), a domain with high sequence variability (V), and two terminal domains (TL and TR) that are interchangeable between viroids (Fig. 2).^27)^

More comprehensive analysis to investigate the role of individual structural domains in viroid pathogenicity and replication using a series of interspecific chimeras constructed by exchanging the TL and/or P domains between tomato apical stunt viroid (TASVd) and CEVd revealed: (i) the TL domain of TASVd contains a determinant required for the presentation of severe veinal necrosis in tomato; (ii) the severe epinasty and stunting characteristic of TASVd requires the presence of the TL and P domains; and (iii) the V and TR domains comprising the right side of the native structure also play an important role in viroid pathogenicity. Although the individual contributions of the TL and P domains to symptom induction were not completely separated from the viroid titer, the TL domain also appears to exert a greater effect upon symptom severity. These results indicated that multiple domains (TL, P, V, and TR) contain sequences and/or structural motifs that correspond to the pathogenicity determinants of pospiviroids in general.^28)^

### 1.2 Structural motifs for replication.

#### Mode of replication—Rolling circle.

The existence of antigenomic (−) strands of a viroid molecule with the same mobility as the linear molecule and with a high molecular weight were reported in plants infected with PSTVd and CEVd, respectively, suggesting their involvement in the processes of replication.^29,30)^ Further evidence that longer-than-unit-length PSTVd (−) strands with size-exact multiples of PSTVd unit-length (*i.e.*, about 700, 1,050, 1,500, and 1,800 nucleotides long) exist in extracts of PSTVd-infected plants led to the proposal of a rolling-circle replication model for (−) strand synthesis.^31,32)^ Briefly, in the case of pospiviroids, replication begins with the transcription of longer-than-unit-length (−) strands from a unit-length circular (+) strand (or genomic strand) via a rolling circle. They then serve as templates, and longer-than-unit-length (+) strands are synthesized. Finally, they are cut into the unit-length and circularized to complete the replication cycle. The model was confirmed by the detection of replicative intermediates containing monomeric, circular, or linear PSTVd strands complexed with multimeric (−) strands^33)^ and using an infectivity assay of tandemly repeated genomic (+)- and (−)-strand hop stunt viroid (HSVd) RNA synthesized *in vitro* from cloned HSVd cDNA.^34)^

#### Host enzymes for replication.

First, replication of viroids belonging to the family *Pospiviroidae* was shown to be inhibited by low concentrations of α-amanitin, a selective inhibitor of DNA-dependent RNA polymerase II (DdRPII),^35)^ and DdRPII purified from healthy plant tissue was capable of synthesizing linear (−)-viroid RNA copies of full length from (+)-viroid RNA templates *in vitro*,^36)^ which suggested that nuclear DdRPII is involved in viroid replication. Further studies on PSTVd synthesis *in vivo* using highly purified nuclei with viroid replicates from suspension cultures of PSTVd-infected *Solanum demissum* cells as an assay system revealed that PSTVd (+)- and (−)-RNA synthesis was inhibited at the same α-amanitin concentrations as for host mRNA, which is transcribed by DdRPII. However, host mRNA transcribed by DdRPI or DdRPIII was not inhibited at the same α-amanitin concentration. Furthermore, PSTVd synthesis was not inhibited by actinomycin D, which interferes with DNA replication by binding to DNA duplexes even at high actinomycin D concentrations, indicating that PSTVd replication does not depend on the active transcription of chromosomal DNA. These results confirmed that the DdRPII is directly involved in PSTVd (+)- and (−)-RNA synthesis.^37)^

#### Transcription start site.

A putative transcription start site was thought to be located within the TL loop, either at nucleotide U359 or C1,^38)^ which was supported by evidence that a DdRPII complex from a natural host interacted with the rod-like conformation of the TL domain of PSTVd (+)-RNA.^39)^

#### Transcription factors for replication.

Participation of transcription factor IIIA (TFIIIA) and ribosomal protein L5 (RPL5) in the synthesis and delivery of PSTVd RNA *in vivo* was suggested. These two factors from *Arabidopsis thaliana* bind PSTVd (+)-RNA *in vitro* with the same affinity as they bind 5S rRNA.^40)^ Canonical 9-zinc finger transcription factor IIIA (TFIIIA-9ZF), as well as its splicing variant TFIIIA-7ZF, interacted with PSTVd (+)-RNA, but only TFIIIA-7ZF was shown to interact with PSTVd (−)-RNA. RNase protection assay data indicated the binding sites were within the TL domain. Suppression of TFIIIA-7ZF reduced PSTVd replication, whereas overexpression of TFIIIA-7ZF enhanced PSTVd replication *in planta*, which indicated that TFIIIA-7ZF is essential for DdRPII to replicate PSTVd.^41)^ PSTVd is thought to downregulate its expression through direct interaction with RPL5, a TFIIIA splicing regulator, and optimizes the expression of TFIIIA-7ZF by facilitating splicing from TFIIIA-9ZF to TFIIIA-7ZF, which is favorable for PSTVd replication.^42)^

#### Central conserved region—Processing site for genomic strand replicative intermediate.

Infectivity assays using longer-than-unit-length cDNA copies or *in vitro* transcripts of some pospiviroids revealed that linear RNA molecules with a short terminal duplication in the upper part of the CCR are highly infectious.^43–46)^ In particular, the direct repetition of the 14-nucleotide sequence (5′-GGAUCCCCGGGGAA-3′) at both ends of the longer-than-unit-length (+)-strand replication intermediate was shown to be able to display full infectivity. In addition, the 11-nucleotide (5′-GGAUCCCCGGG-3′) sequence alone exerted strong infectivity, but the infectivity was reduced by tenfold.^47,48)^ Based on these data, CCR was proposed as an essential site for viroid replication as a processing site of the oligomeric forms of (+)-strand replication intermediates into monomers.^49)^ The cleavage-ligation site was mapped between G95 and G96 in the CCR, and the conformational change from a GAAA tetraloop to loop E in the region was shown to drive the switching from cleavage to ligation (Fig. 2).^50–52)^ The loop E motif is apparent in a variety of different RNAs such as 16S and 23S rRNA, group I and II introns, RNase P, and the hairpin ribozyme, which is highly structured by non-canonical base pairs and is involved in RNA–RNA as well as RNA–protein interactions.^52)^ PSTVd (+)-strand RNA was also reported to form loop E *in vivo*, suggesting that this structural motif is involved in a wide range of functions such as replication, host specificity, and pathogenesis.^53–55)^ Furthermore, an *in silico* survey showed that members of the *Pospiviroidae* family can form a motif that resembles a sarcin/ricin domain or loop E in their CCR and implied that there could be undiscovered mimics of RNA domains to recruit host factors.^56)^

### 1.3 Structural motifs regulating pathogenicity.

#### Pathogenicity domain—Thermal instability of pre-melting (PM) loop in the VM region.

Extensive research has been conducted to identify the RNA structural features (or motifs) responsible for viroid pathogenicity. In early studies in the mid-1980s, the P domain was shown to play a critical role in modulating the virulence of PSTVd.^23)^ In that study, nucleotide sequence, thermodynamic, and pathogenicity analyses of four natural PSTVd variants with different pathogenicities (KF6, DI, HS, and KF440 in order of mild to severe) showed that the thermodynamic instability of the PM loop 1 in the VM region within the P domain was correlated with virulence (Fig. 2). In this scenario, it was thought that thermodynamic instability in the region of PM loop 1 may facilitate the binding of unidentified host factor(s), and as a result, would specify and regulate the virulence of PSTVd. Comparison of the nucleotide sequences of another set of mild (KF5) and severe (S) variants of PSTVd also provided support for the hypothesis that increasing thermodynamic instability of the VM region is correlated with increasing virulence of the respective naturally occurring PSTVd variant.^57)^ Comparative analysis of the lethal-type PSTVd mutant RG1 and intermediate-type mutants QF-A and QF-B that emerged spontaneously from an intermediate strain (DI, or the Diener’s isolate) during incubation in a greenhouse revealed that the mutations in these variants (three in RG1, two in QF-A, and three in QF-B) compared with those of DI were all located in the VM region. These mutations affected the thermal instability of the VM region and correlated with the virulence of the variants, findings that supported the hypothesis. An advantage in replication and a beneficial transient structure, which is active in replication, was also assumed to be essential in pathogenesis.^58)^

The generation of mutants using site-directed mutagenesis to replace the four substitutions at nucleotides 46, 47, 315, and 317 in the VM region of the intermediate and severe strains demonstrated that a previously proposed correlation between thermodynamic instability and PSTVd pathogenicity did not hold true in all cases.^59)^ In mutational analysis by reverse genetics using an infectious PSTVd cDNA clone, mutations that stabilized the P domain (*i.e.*, the PM loop 1 and VM region) *in vitro* suppressed symptom expression. However, the conformational stability appeared to be only one of several factors regulating PSTVd replication and pathogenicity.^60)^ Infectivity studies using six intraspecific PSTVd chimeras constructed by exchanging the P and V domains between one mild and two severe PSTVd variants showed that the P domain is directly responsible for the severity of symptoms in tomato. Still, symptom severity did not always correlate with viroid accumulation, indicating that the impact on the development of symptoms through the P domain is not straight-forward. We should also consider the potential contribution of the V domain.^61)^

Research on nucleotides involved in the attenuation of PSTVd has also progressed. The dahlia isolate of PSTVd-D accumulates slowly and induces mild disease symptoms in tomato (cv. Rutgers) compared with the intermediate strain. The dahlia isolate differs from the intermediate strain at nine nucleotides located in the TL, P, V, and TR domains.^62)^ A series of mutational analyses followed by infection assays revealed that two mutations at positions 42 and 64 in the TL and P domains significantly reduced the virulence and accumulation of the viroid (Fig. 2).^63–66)^ In particular, a C42U mutation significantly impaired PSTVd replication and did not elicit excessive host defense responses, thus contributing to the attenuation of disease symptoms. Although mutations at sites 310–312 in the VM region did not play a significant role in pathogenicity, these nucleotides, in cooperation with nucleotides 42, 43, and 64 in the paired strand, contributed to replication of the dahlia isolate by maintaining the local structure of the TL-P domains (Fig. 2).^66)^

#### Bending of the molecule within the pathogenicity domain.

Mutational analysis also indicated that in certain cases, downregulation of pathogenicity was correlated with suppression of the rate of PSTVd replication/accumulation. However, there was no consistent correlation between symptom severity and thermodynamic instability of the VM region. Rather, a comparison of the optimal secondary structures of these mutants, taking into account the three-dimensional shape of the RNA helix, showed that there were significant differences in the geometry of the shape of their P domains. That is, the mutant that caused intermediate symptoms possesses a linear arrangement of three consecutive helices, whereas the variant that caused mild or severe symptoms harbors a domain that is bent in the opposite direction. These results suggested that alterations in the RNA structure and concomitant alterations in RNA–protein interaction(s) may be the primary cause of viroid pathogenicity.^67)^ This hypothesis was verified experimentally using *in vitro* transcribed model RNAs and gel mobility assays in the presence or absence of Mg^2+^ to determine whether slight changes in the nucleotides of the VM region result in varying degrees of bending of this part of the molecule based on the notion that minor sequence variations in the VM region of the P domain can distinguish strains that cause mild disease from those that cause severe and even lethal disease. A striking correlation was observed between bending of the VM region and the pathogenicity of the respective variants, except for one variant whose thermodynamically stable structure was kinked more strongly than would be expected from its observed pathogenicity. However, the suboptimal structure of the exceptional variant fit perfectly into the bending versus pathogenicity scheme, supporting the hypothesis.^68)^

#### Central domain.

As described for replication, the C domain plays an important role as a processing site for (+)-strand replication intermediates and contains structural motifs involved in a wide range of functions such as host specificity and pathogenesis. Mechanical inoculation of *Nicotiana tabacum* with the PSTVd variant KF 440-2 from a host tomato plant resulted in *de novo* emergence, replication, and accumulation of a new tobacco variant designated PSTVd-NT.^69)^ Sequence analysis revealed a single C-to-U nucleotide substitution at position 259, which is in loop E (Fig. 2). A C259U change might affect host specificity through a conformational change in the loop E structure. A U257A change, but not a C or G change, in the CCR converted the intermediate strain of PSTVd-I to a lethal strain that caused severe stunting and premature death of infected plants.^70)^ The stunted growth of the infected tomato plants resulted from restricted cell expansion but not cell division or differentiation. It was correlated positively with the downregulated expression of an expansion gene, LeExp2. The U257A substitution did not alter the PSTVd secondary structure, replication levels, or tissue tropism. A C259U substitution abolished the pathogenic effect of the U257A substitution in the same RNA molecule.^70)^

### 1.4 Structural motifs for cell-to-cell movement and systemic trafficking.

After invading epidermal cells, viroids are transported to the cell nucleus or chloroplasts, where they replicate before exiting to the cytoplasm and moving from cell to cell via the plasmodesmata of the mesophyll to the bundle sheath and then to the phloem in the inoculated leaf. The viroids traffic long distances in the phloem and across cellular boundaries to spread into the upper leaves. PSTVd movement from cell to cell via plasmodesmata and long-distance trafficking across cellular boundaries in the phloem is mediated by a specific sequence or structural motif in its RNA.^71,72)^ The predicted secondary structure of viroid RNA contains many loops and bulges flanked by double-stranded helices (Fig. 2). These loops and bulges are functional motifs that regulate replication in single cells or systemic trafficking in a plant; in the case of PSTVd, 11 out of 27 loops were identified as motifs critical for systemic trafficking (Fig. 2).^73)^ More specifically, loop 6^74)^ and loop 19^75,76)^ were identified as RNA motifs required for PSTVd movement from the palisade mesophyll to the spongy mesophyll in *Nicotiana benthamiana*. Loop 7, consisting of U43/C318, was identified as critical for systemic trafficking from the bundle sheath to the phloem.^77)^ A bipartite trafficking motif consisting of U201 in loop 24 in the TR domain and U309 and U47/A313 in the P domain was shown to be necessary and sufficient to mediate the trafficking of a PSTVd-NB variant from the bundle sheath to the mesophyll in tobacco.^78)^ Recently, loop 27 of PSTVd was shown to be required for epidermal exit, but not epidermal entry or transit, between other cell types.^79)^

Meanwhile, a comprehensive mutational analysis demonstrated that nearly all G/U pairs, but not loops, are critical for replication and systemic spread.^80)^ Among them, the following seven G/U pairs appeared to be essential for infectivity: one G/U pair formed by nucleotides 7 and 353 (7:353) for replication, four pairs by 27:335, 44:317, 61:299, and 156:205 for systemic spread, and two pairs by 64:296 and 76:283 for entry into, but not exit from, the host vascular system.^80)^ It was noted that the 7U:353G pair lies within a region known to bind DdRPII and its cofactor TFIIIA-7ZF, which together transcribe (+)-PSTVd RNA to initiate the replication process.

A protein of 602 amino acids containing a bromodomain, termed viroid RNA-binding protein 1 (VirP1), was identified from a tomato cDNA expression library by investigation of RNA-binding proteins that specifically interact with PSTVd.^81)^ VirP1 strongly and specifically interacted with the TR domain of PSTVd, while weakly interacting with the TR domain of HSVd, which replicates less aggressively in tomato plants.^82)^ It has been suggested that the AGG/CCUUC motif forming parts of loops 24 and 26 within the TR domain enhances the recognition of TR by VirP1, and the motif could be involved in the systemic transport of viroids.

### 1.5 Functions of viroid-specific small RNAs.

Potato spindle tuber viroid induces strong RNA silencing in infected tomato plants, and the infected plants accumulate short RNA fragments called “viroid-specific small RNAs (vd-sRNA)” characteristic of post-transcriptional gene silencing.^83–85)^ Accumulation of vd-sRNA was reported in other pospiviroid-infected plants such as HSVd-infected cucumber (*Cucumis sativus*), grapevine (*Vitis vinifera*), and hops; hop latent viroid (HLVd)-infected hops; apple fruit crinkle viroid (AFCVd)-infected hops and tomato; CEVd-infected tomato; citrus bark cracking viroid (CBCVd)-infected hops; coleus blumei viroid 1, 5, and 6 (CbVd-1, 5, 6)-infected coleus^86–92)^; and avsunviroid-infected plants such as peach latent mosaic viroid (PLMVd)-infected peach, chrysanthemum chlorotic mottle viroid-infected chrysanthemum, and avocado sunblotch viroid (ASBVd)-infected avocado.^93,94)^ In pospiviroids that replicate in the nucleus, the major classes of vd-sRNAs are 22 and 21 nt in size and accompanied by significant levels of 24-nt species.^89)^ In avsunviroids that replicate in chloroplasts, the 21-nt class is predominant, followed by the 22-nt class, but the 24-nt class is negligible.^94,95)^ These vd-sRNAs are not uniformly produced from the entire viroid genome but are produced in particularly large amounts from specific hotspot regions of each of the genomic [or (+)] and antigenomic [or (−)] strands. In PSTVd-infected tomato plants, PSTVd-specific small RNA (PSTVd-sRNA) hotspot patterns differ between susceptible and tolerant cultivars,^96)^ and a severe PSTVd variant produces more PSTVd-sRNAs than a mild variant of the P domain.^65)^

**Figure 3.**
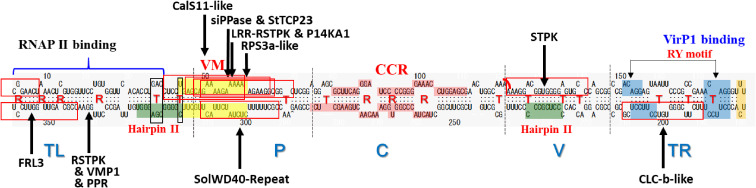
(Color online) A genetic map of PSTVd with vd-sRNAs that have the potential to inhibit host gene expression. The sequences in the red frames indicate the regions that gave rise to vd-sRNA, which may inhibit host gene expression. Words with arrows indicate target candidate genes. Other structural domains and important motifs are similar to those in Fig. 2.

A viroid is an infectious RNA molecule that does not encode a protein, suggesting that vd-sRNA produced by host RNA silencing defenses may be involved in the pathogenic processes of viroid infection and the evolution of viroids.^97)^ This hypothesis was substantiated by a contagious peach disease called peach calico (PC) characterized by extreme chlorosis of leaves and caused by PLMVd infection.^98)^ PLMVd-sRNAs (PC-sRNA8a and PC-sRNA8b) derived from the PC-associated insertion of a specific PLMVd variant that causes PC are able to target and cleave the mRNA encoding chloroplastic heat-shock protein 90 (cHSP90), resulting in the inhibition of the formation of chloroplasts, leading to the elicitation of PC symptoms. In addition, many vd-sRNAs with the potential to inhibit expression of certain host genes have been reported in *Pospiviroid*, mainly in PSTVd and solanaceous host plant combinations such as tomato, tobacco, and potato.^64,99–104)^ Of note, many of these PSTVd-sRNAs mapped to the P domain (Fig. 3). These vd-sRNAs, as functional RNA fragments produced from a non-coding viroid RNA genome, may act like a small interfering RNA (siRNA), miroRNA (miRNA), or a trans-acting siRNA (ta-siRNA) to inhibit or control expression of a host gene containing the target sequence, leading to disease symptoms.^105)^ Alternatively, large quantities of vd-sRNA produced in infected plants may adversely affect host development or morphogenesis through indirect interference with the host siRNA/miRNA pathways.^106)^ The role of RNA silencing in triggering the initial molecular lesion has not yet been fully clarified, particularly in the pospiviroids^107)^; however, the potential pathogenic effect of vd-sRNAs that accumulate in large quantities in infected plants is an interesting issue worthy of further consideration.

### 1.6 Structural motifs in other pospiviroids.

In pospiviroids other than PSTVd, structural domains and motifs related to pathogenicity, seed transmission, or pollen transmission have also been elucidated, although knowledge is fragmented. Citrus viroid (CVd) group II is composed of HSVd-related variants of 295–302 nucleotides in length, including the cachexia-inducing variants CVd-IIb, CVd-IIc, Ca-903, and Ca-909 and the non-cachexia-inducing variants more similar to the hop-type of HSVd called CVd-IIa. The cachexia-inducing variants produce symptoms of gumming, discoloration, and stem pitting on sensitive citrus varieties ‘Parsons’ Special’ mandarin, ‘Orlando’ tangelo, and ‘Palestine’ sweet lime. These variants exclusively contain five to six nucleotide substitutions consisting of A107G, A109del, A115del, U189del, U194C, and C197U in the “cachexia expression motif” of the upper and lower strands of the V domain.^108,109)^ Site-directed mutagenesis studies further showed evidence that a single-nucleotide change can modulate the expression of cachexia symptoms; the motif plays a major role in inciting cachexia symptoms, and subtle changes within this motif affect symptom severity.^110)^ Specifically, among the nucleotides in the motif, the one at position 197 is particularly interesting. Comparison of two HSVd-cachexia variants, CVd-IIa and CVd-IIb, which differ by a single C/U substitution at position 196 corresponding to position 197 of the aforementioned variants, revealed that CVd-IIa with a C in position 196 caused more severe symptoms in sponge gourd (*Luffa aegyptiaca*) than CVd-IIb with a U at position 196.^108)^ Similarly, in an independent comparative analysis using the grapevine variant of HSVd and its hop-adapted mutants (see next section), the mutant with a C at position 193 caused more severe symptoms in cucumber than that with a U.^111)^ Because nucleotide 193 of the HSVd-grapevine variant corresponds to nucleotide 197 of CVd cachexia-inducing variants, nucleotide 197 or 193 in the V domain appears to be an important pathogenicity determinant in HSVd, at least in cucurbits.

Tomato planta macho viroid (TPMVd), also known as Mexican papita viroid, causes serious disease in tomato plants, and only one base-pair mutation in the TR domain (*i.e.*, 176U:A185 in the mild variant and 174G:C183 in the severe variant) was identified as a virulence determinant factor.^112)^ In addition, the sequences or structures in the TL and P domains of TPMVd involve a major determinant for horizontal and vertical transmission in petunia plants by pollen.^113)^ Nucleotide 25 in loop 5 in the TL region was identified as a determinant for seed transmission of CbVd-1, known for an extremely high seed transmission rate.^114)^ Meanwhile, in an experiment of inter-subgroup chimeras in which the TR domain was exchanged between CEVd and HSVd, the TR domain could be exchanged while maintaining infectivity, but the accumulation in the hosts, tomato and cucumber, was reduced 10-fold or more. The results suggested that the TR domain forms a relatively independent structural unit that contributes to modulate the efficiency of viroid replication or accumulation.^115)^

### 1.7 Short summary—Current overviews of structural motifs regulating pospiviroid pathogenicity.

Among the five structural domains proposed for the members of the family *Pospiviroidae*, the major determinants associated with the pathogenicity were mapped to the P domain and attributed to the thermodynamic instability of PM loop 1 in the VM region. The bending angle of the native structure in the VM region was completely correlated with pathogenicity. The findings suggest that the P domain is a region that recruits and interacts with still unidentified host factors involved in replication, cell-to-cell movement, and long-distance transport. Furthermore, among the vd-sRNA generated from various regions of the molecule, a considerable number derived from the P domain were shown to have the potential to target and inhibit host gene expression via RNA silencing mechanisms. The pathogenic effects of vd-sRNA need further analysis. The molecular determinants that regulate replication were mapped to the TL and C domains. A putative transcription start site was thought to be within the TL domain, probably at the left end portion, and a binding site for the transcription factor TFIIIA-7ZF was also identified within the TL domain. The CCR in the upper strand of the C domain was shown to be a processing ligation site for the production of the unit-length from longer-than-unit-length (+)-strands at the final stage of replication. Because replication is an important factor in determining pathogenicity and is keenly associated with the elicitation of host defense responses, as will describe later in this review, the C domain is a region that regulates pathogenicity. Moreover, loops and G/U pairs scattered throughout the molecule are also critical for replication and systemic spread. In conclusion, from analysis of a wider range of pospiviroid members such as PSTVd, CEVd, HSVd, TASVd, TPMVd, AFCVd, and CbVd-1, it has generally been established that multiple domains including TL, P, V, TR, and C possess sequences and structural motifs that contribute to pospiviroid pathogenicity (Fig. 2).

## 2 Host responses against viroid infection

Although a lot of knowledge has been accumulated about the functional motifs and molecular structures of viroid genomic RNA, the many host factors that interact with them remain unclear. For example, how do systemic symptoms most typical of viroid infection, such as dwarfing, leaf malformation, yellowing, and necrosis, occur? What host factors are involved in the development of disease symptoms? Comprehensive genome-wide analyses of gene expression in several host plants afflicted by various viroid infections are in progress.

### 2.1 Genome-wide fluctuations in gene expression.

Comprehensive analysis of the differential gene expression patterns of viroid-infected plants was performed for the first time in tomatoes infected with mild and severe strains of PSTVd using macroarrays containing 1,156 clones from the subtracted tomato cDNA library.^116)^ The analysis revealed that the two PSTVd strains altered the expression of both common and unique tomato genes, and a total of 55 differentially expressed genes in tomato unique to PSTVd infection were identified, including those associated with defense/stress response, cell wall structure, chloroplast function, and protein metabolism. Global analysis of tomato gene expression during PSTVd infection by microarray analysis then revealed that the mRNA expression levels of more than half of the approximately 10,000 genes present on the array were altered in the susceptible tomato cultivar “Rutgers”,^117)^ including: downregulation of chloroplast biogenesis; changes in mRNAs encoding enzymes involved in the biosynthesis of gibberellin and other hormones; and a marked upregulation of genes involved in response to stressors and other stimuli. More recently, comprehensive transcriptome analyses by RNA sequencing or RNAseq revealed that PSTVd triggers genome-wide changes in alternative splicing or inducible trans-acting activity of phased secondary siRNAs, and massively activates genes involved in plant immune responses, mainly those in the calcium-dependent protein kinase and mitogen-activated protein kinase (MAPK) cascades, as well as prominent genes involved in hypersensitive responses, cell wall fortification, and hormone signaling.^118)^ A time-course analysis by microarray analysis of the changes in gene expression in “Rutgers” tomato leaves in response to the mild (M) and severe (S23) variants of PSTVd revealed that over 3,000 genes were affected in plants infected with the S23 variant, whereas those infected with the M variant showed three-fold fewer affected genes. The differentially expressed genes included many related to: stress; defense; hormone metabolism and signaling; photosynthesis and chloroplasts; the cell wall; RNA regulation, processing and binding; and protein metabolism and modification.^119)^

Similar analyses on viroid–host interactions were performed for combinations other than PSTVd and tomato. Initiation of a complex array of changes in the host transcriptome by viroid infection was reported in cucumber plants infected with a severe and mild HSVd variant, in which many genes related to photosynthesis were downregulated. Those encoding RNA-dependent RNA polymerase 1 (RdRP1) and related to basal defense responses were upregulated. The expression levels of genes associated with phytohormone signaling pathways were also altered.^120)^ Induction of these responses occurs earlier and is stronger in plants infected with a severe variant (HSVd-g54) than in those infected with a mild variant (HSVd-h).

Comprehensive transcriptome analyses of hops infected with CBCVd alone or co-infected with HLVd revealed that CBCVd infection resulted in the massive modulation of the activity of over 2,000 genes, and a mixed-type infection may result in more significant interference with host factors. Expression of genes associated with plant immune responses such as: protein kinase and MAPK; hypersensitive responses; phytohormone signaling pathways; photosynthesis; pigment metabolism; protein metabolism; and sugar metabolism and modification were altered.^121,122)^ In addition, genes encoding RdRP, a pathogenesis related (PR) protein, chitinase, and those related to basal defense responses were upregulated.

Global transcriptomic analysis on CEVd-infected “Etrog” citron detected 1,530 differentially expressed genes involved in the RNA silencing pathway such as *dicer like-2* (*DCL2*), *RdRP1*, *argonaute 2* (*AGO2*), *AGO7*, and *silencing defective 3*, as well as those genes encoding proteins that are related to basic defense responses. Many genes involved in secondary metabolite biosynthesis and chitinase activity were upregulated, whereas other genes related to the cell wall and phytohormone signal transduction were downregulated. Moreover, genes encoding disease resistance proteins, PR proteins, and the heat-shock cognate 70 kDa protein were upregulated in response to CEVd infection. These results suggested that basic defense and RNA silencing mechanisms are activated by CEVd infection.^123)^

The induction of global fluctuations in gene expression in chrysanthemum infected with chrysanthemum stunt viroid (CSVd) was reported, in which the expression levels of genes involved in the biosynthesis of gibberellic acid (GA) and cytokinin (CK), indole-3-acetic acid (IAA) transport, and cell wall growth in infected plants were downregulated, thus suggesting that downregulation of GA responsiveness and inhibition of cell wall expansion are major causes of the stunted growth of CSVd-infected chrysanthemum plants.^124)^

### 2.2 Upregulation of RNA silencing factors.

As described above, one of the most intriguing changes in genome-wide gene expression in viroid-infected plants is the upregulation of key factors in RNA silencing, such as *DCLs*, *AGOs*, and *RdRP6*. Because these RNA silencing factors are known to function at the forefront of defense mechanisms against foreign invaders like viruses and viroids, it was not surprising to expect that the highly base-paired stem–loop structure of viroid RNA would be the substrate for multiple DCLs. In fact, direct evidence for such cleavage was first obtained from experiments showing that PSTVd RNA was cleaved into small pieces approximately 21 nt in length when incubated with an *Arabidopsis* cell extract possessing DCL activity.^85)^ In a PSTVd infection experiment against a series of transgenic *N. benthamiana* lines in which all four *DCL* genes and their combinations were knocked down, the accumulation of PSTVd was shown to drop when *DCL4* gene expression alone was suppressed and when the *DCL1*, *DCL2*, or *DCL3* genes were knocked down along with the *DCL4* gene.^125,126)^ These observations led to a hypothesis that the combined activity of DCL2 and DCL3 is crucial in the defense against PSTVd, in which DCL4 is supposed to play a key role in the processing PSTVd RNA; its activity can mask or suppress the effects of DCL2 and/or DCL3, suggesting that hierarchical interactions exist among DCLs in defense against viroids. In the PSTVd-tolerant tomato cultivar “Moneymaker”, simultaneous knockdown of *DCL2* and *DCL4* genes also increased susceptibility to PSTVd, indicating that these DCLs play an important role in the defense against viroid infection.^127)^

Using PSTVd-infected *N. benthamiana*, it was shown that AGO1, AGO2, AGO3, AGO4, AGO5, and AGO9 have been associated with vd-sRNAs, and each AGO has a preference for vd-sRNA based on the 5′-terminal nucleotide sequence and size, similar to that shown in viruses.^128)^ In addition, overexpression by agroexpression of the endogenous or exogenous AGO1, AGO2, AGO4, and AGO5 reduced the accumulation of PSTVd, supporting their role in antiviroid defense.

Regarding RdRPs, RdRP1 expression was found to be enhanced in PSTVd-infected “Rutgers” tomatoes and HSVd-infected “Suyo” cucumbers, suggesting the involvement of RdRP1 in antiviroid defense.^120,129)^ In addition to RdRP1, the role of RdRP6 in plants infected with viroids was first analyzed using RdRP6-knockdown *N. benthamiana* plants infected with HSVd.^130)^ In this report, the scion of RdRP6-knockdown *N. benthamiana* remained asymptomatic when grafted onto symptomatic plants, despite an accumulation of a high level of HSVd, indicating the requirement of RdRP6 for viroid-induced symptom expression. On the other hand, for a PSTVd–*N. benthamiana* combination, the accumulation of PSTVd in the early infection stage was increased, and invasion by PSTVd into floral and vegetative meristem was promoted in RdRP6-knockdown *N. benthamiana* compared with that of control plants,^131)^ suggesting that RdRP6 is involved in restricting the systemic spread of viroids and precluding their invasion of the apical growing tips. Similarly, suppression of RdRP6 in *N. benthamiana* using virus-induced gene silencing resulted in an approximately three-fold increase in the accumulation of PSTVd RNA compared with that seen in the control plants, suggesting that a direct correlation exists between RdRP6 and viroid accumulation.^132)^ The result was consistent with a previous observation by Di Serio *et al.* (2010)^131)^ and indicated that *N. benthamiana* RdRP6 functions to suppress PSTVd accumulation. Moreover, the accumulation of PSTVd in the early infection stage in a PSTVd–tomato combination was significantly lower in an RdRP6-knockdown “Moneymaker” tomato compared with that of the control plants; invasion into the apical meristem was, at most, up to the basal portion even in an RdRP6-knockdown tomato and did not reach the apical portion of the shoot apical meristem (SAM), unlike was reported in *N. benthamiana*.^133)^ Considering that the *N. benthamiana* derivative used was a natural loss-of-function variant of NbRdRP1,^134)^ these results suggested host factors other than RDRP6 (like RdRP1) may be involved in the protection of SAM and pluripotent stem cells from PSTVd invasion.

### 2.3 Fluctuation of microRNA biogenesis.

As described above, global transcriptome analysis and small RNA sequencing revealed that the levels of various miRNA species, including miR159, miR171e, and miR4376, fluctuated in viroid-infected plants.^63,117,118)^ High-throughput sequencing analysis of hops infected with CBCVd enabled the identification of a total of 67 conserved and 49 novel miRNAs; 36 conserved and 37 novel miRNAs were found to be differentially expressed in response to CBCVd infection. These miRNAs were predicted to have a total of 311 potential targets, the majority of them being transcription factors that may regulate hop leaf, root, and cone growth and development. In addition, the identified miRNAs may play important roles in other cellular and metabolic processes, including signal transduction, stress response, and physiological processes such as the prenylflavonoid biosynthesis pathways.^106)^ Among the miRNAs induced by viroid infection, miR398 and miR398a-3p were particularly interesting. RNAi-mediated knockdown of the *DCL2* and *DCL4* genes, key factors in RNA silencing, was reported to make a PSTVd-tolerant tomato cultivar “Moneymaker” highly susceptible to PSTVd, causing it to present with severe systemic necrosis.^127)^ Therein, the expression of the stress-responsive miR398 and miR398a-3p increased approximately 8-fold, and the expression levels of cytosolic and chloroplast-localized Cu/Zn-superoxide dismutase 1 and 2 (SOD1 and SOD2) and the copper chaperone for SOD (CCS1) mRNA, potential targets of miR398 and 398a-3p, decreased significantly. The same was observed with the PSTVd-sensitive tomato cultivar “Rutgers”.^135)^ These results suggested that a high level of expression of miR398 and miR398a-3p induced by viroid infection disrupts functioning to regulate the expression of *SOD* genes and impairs control of excessive ROS production, leading to the development of lethal systemic necrosis.

### 2.4 Activation of double-stranded RNA-dependent protein kinase.

A host-encoded protein of Mr 68,000 (P68), which was differently phosphorylated in infected tissues, was identified in an extract from a viroid-infected plant, and the phosphoprotein was immunologically related to a double-stranded RNA-dependent protein kinase.^136)^ PSTVd-activated (*i.e.*, phosphorylated) protein P68 and activation by the severe strain was at least ten times higher than that of the mild strain, although the mild and severe strains differed by only a two-nucleotide inversions (UUC to CUU) in the lower portion of the P domain.^137)^ P68 is termed pPKR and was shown to be an analog of mammalian PKR enzymes. pPKR is both cytosolic and ribosome-associated, similar to mammalian PKR. It appears to be capable of phosphorylating exogenous histones.^138)^ Another protein kinase gene, pkv, was identified in PSTVd-infected tomato plants, in which the level of transcriptional activation was higher in plants infected with the severe and intermediate variants than those with the mild variant. A full-length copy of the gene encoding the 55 kDa protein kinase viroid-induced protein was isolated, and sequence analysis revealed significant homology with cyclic nucleotide-dependent protein kinases.^139)^

### 2.5 Ribosome stress.

“Ribosomopathies” are human diseases associated with ribosome dysfunction normally due to a defect in ribosome biogenesis, and “ribosomal stress” induces cell-cycle arrest and apoptosis in the affected cells and tissues.^140)^ This pathway seems to be absent in the plant kingdom. However, infection with CEVd was shown to cause ribosomal stress in infected tomato plants, presenting as alterations in the polysome profiles, ribosomal RNA processing (especially in the external transcribed spacers of rRNA), and translational profiles. These alterations were in association with the degree of CEVd symptomatology and with the induction of the ribosomal stress mediator SlNAC82 in infected tomato leaves, supporting the idea that CEVd provokes alterations in the biogenesis of ribosomes in tomato, thereby interfering with the translation machinery.^141)^

### 2.6 Short summary—Host responses of gene expression against viroid infection.

**Figure 4.**
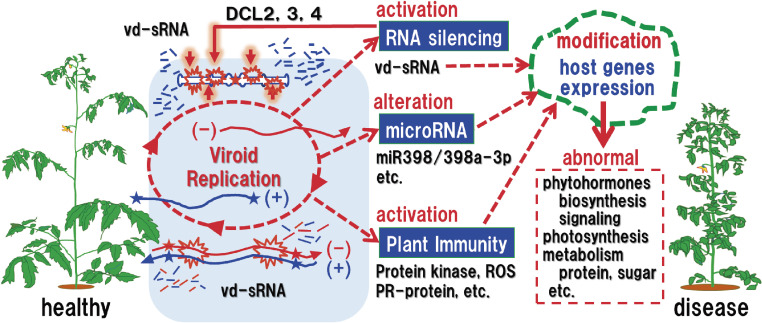
Overview of host defense responses against viroid infection and molecular mechanisms of pathogenicity. Replication/accumulation of viroid RNAs induces RNA silencing targeting viroids, and large amounts of vd-sRNAs accumulate in infected plant cells. These vd-sRNAs disturb normal regulation of host RNA silencing and miRNA pathways. In addition, some of the vd-sRNAs are incorporated into RNA silencing pathways and inhibit the expression of host genes containing complementary sequences. Viroid infection also stimulates plant innate immunity and induces various defense responses, which result in the alteration of numerous host gene expressions, for example, those related to phytohormones metabolism.

Comprehensive analyses of fluctuations of genome-wide gene expression caused by viroid infection, in any viroid–host combinations examined, revealed marked upregulation of genes involved in responses to stress and stimuli and those related to defense responses such as RNA silencing and plant innate immunity, including calcium-dependent protein kinase, MAPK, hypersensitivity responses, PR proteins, and cell wall strengthening. Disruption of miRNA biogenesis was also remarkable. These defense responses against viroid infection cause numerous alterations in gene expression levels. Differentially expressed genes include those related to: metabolism and signaling of phytohormones such as gibberellin; photosynthesis and chloroplast biogenesis; RNA regulation; pigment or protein metabolism and modification; and sugar metabolism. It is quite reasonable to understand that these changes were more strongly incited by infection with more virulent variants (Fig. 4).

## 3 Ecology of viroids—Diversity and host adaptation

Viroids, known primarily as the smallest infectious agent of plants, are likely to be “living fossils” that survived from the pre-cellular RNA world.^10)^ As demonstrated by ASBVd or PLMVd, it has been suggested that the RNA self-cleavage activity prominent in the ribozyme motif of the members of avsunviroid may reflect the characteristics necessary to overcome obstacles in the autonomous replication of primitive RNAs. The discovery of diverse viroid species and the detection of numerous sequence variants revealed that viroids can change the nucleotide sequence in their genome and produce diverse mutants in different environments (*i.e.*, host species, temperature conditions, and geographical isolation). They have one of the fundamental attributes of life by being able to adapt to the environment according to Darwinian selection. The latter half of this review focuses mainly on HSVd, which is known to have a diverse host range and various mutants, in order to introduce progress in research on viroid diversity, host adaptation, and the ecology behind the emergence of new diseases.

### 3.1 History and origin of hop stunt disease.

#### Emergence of hop stunt disease.

Hops are native to the Caucasus region on the Mediterranean coast and are one of the raw materials used for brewing in Europe (present southern Germany) since the mid-8th century. In Japan, Western varieties were introduced from the United States and Germany and were cultivated for the first time in the mid-1870s (early *Meiji* era) with the start of domestic beer brewing.^142,143)^

In about 1952, poorly growing “dwarf hops” with low overgrowth and short internodes were noticed locally in the *Aizu* region of Fukushima Prefecture and attracted attention as a growth disorder or a new disease.^144,145)^ The buds of the severely infected plants were light reddish and turned green as they grew. The vines were green and thin, the leaves spread and opened earlier, the internodes were shortened from about 1 m in height, and the main foliage was small and brittle, rolling downwards and yellowish. In surveys that started in 1965, the length of the main vine of severely infected plants was reduced to half or less than that of the healthy plants. The yield per acre decreased from 219 kg in healthy fields to 148 kg (67.6% of healthy fields) in severely infected fields in which 60–100% of plants were infected. The cones of the infected hops became elongated and slightly smaller. Analysis of resin content revealed that α-acid content in the cones decreased from 6.6% in the healthy plants to 2.8% in the diseased plants; β-acid content decreased from 5.8% to 5.0%, and the α/β ratio from 1.14 to 0.56, indicating that α-acid content was significantly decreased in diseased cones. The disorder, later named “hop stunt disease (HSD)”, has been scattered in established yards in the region since about 1952 and was rumored to have been observed prior to this. According to the hop cultivation history in the area, the pioneer hop stocks were originally obtained from yards in Yamanashi Prefecture in 1940–1941. The outbreaks began around 1948 in one of the oldest yards, and others became established afterward. Investigation on the infection source revealed that cuttings were a possible carrier of the disease, and all cuttings from infected yards were supplied for planting around 1945–1949 directly or indirectly from one of the pioneer yards (*i.e.*, the index case in the epidemic) established in 1941. Hops showing similar symptoms were also recognized in some yards in the *Tozama* district of *Iiyama* City (about 160 km away from *Aizu*) in Nagano Prefecture, where they were called *Tozama* disease or cedar tree-shaped hops. It was thought to be the same pathogen as that causing HSD in Fukushima. The number of infected yards expanded further and became an epidemic throughout the *Tohoku* region, the main hop production area in Japan from the 1960s to the 1980s. According to a survey in 1967, of the total area of 21,713 acres in the *Aizu* jurisdiction, 19,723 acres were surveyed, and the number of diseased yards with an incidence rate of 60% or more was 60 (402 acres that occupied 2.0% of the total), those with an incidence rate of 30–60% was 37 (282 acres, 1.4%), and those with an incidence rate of 30% or less was 232 (2,280 acres, 11.6%). The diseased hops were seen in 381 yards, which accounted for 15% of the total areas in the jurisdiction. The epidemic caused devastating damage to domestic hop cultivation. HSD was a new disease that had never been recorded in the United States or European countries, where hop cultivation began for the first time more than 1200 years ago.

The causal agent of HSD was identified as a new viroid in 1977 and was named hop stunt viroid.^146,147)^ The discovery of HSVd largely relied upon the finding that cucumber is highly susceptible to the HSD agent. The use of a highly sensitive cucumber cultivar “Suyo” as an indicator plant for HSD assays made it possible to carry out a more detailed analysis of the properties of the HSD agent.^146,147)^ Until then, the diagnosis of HSD relied only on visual inspection of hop plants, but the problem was that asymptomatic infection could not be detected due to the years-long incubation period of the HSD agent in hops. The development of a cucumber bioassay has significantly reduced the time required for diagnosis, enabling the detection of HSD-infected hops in the yard faster and more accurately and has greatly contributed to control of the epidemic.^148)^ The cucumber bioassay for HSVd was further replaced by more rapid and reliable molecular assays, including nucleic acid hybridization,^149)^ reverse transcription-polymerase chain reaction,^150)^ and reverse transcription recombinase polymerase assay, which accommodate large sample numbers.^151,152)^

#### Diversity of hop stunt viroid in various host species.

As is always asked about any newly emerging infectious disease, why did hop stunt unprecedentedly, rather than in advanced hop cultivating countries, emerge in Japan, where hop cultivation began just half a century before? Comparative analysis was performed on the pathogenic and physicochemical properties of HSVd and cucumber pale fruit viroid (CPFVd), a causal agent for cucumber pale fruit disease reported in 1974 in the Netherlands.^153)^ The result revealed that they share a very similar host range and pathogenicity.^154,155)^ The complete nucleotide sequence of HSVd was established (HSVd-h, accession X00009).^156)^ The covalently closed single-stranded circular HSVd RNA consisted of 297 nucleotides and formed an extended rod-like structure characteristic of viroids. Analysis of the complete nucleotide sequencing revealed that CPFVd consisted of 303 nucleotides, which is six nucleotides longer than HSVd, and CPFVd shows about 95% overall pairwise sequence identity with HSVd; they are different in 18 positions, including 12 nucleotide exchanges and six insertions.^157)^ They are variants of the same species, and CPFVd was considered a cucumber variant of HSVd (HSVd-cucumber; HSVd-c).

**Figure 5.**
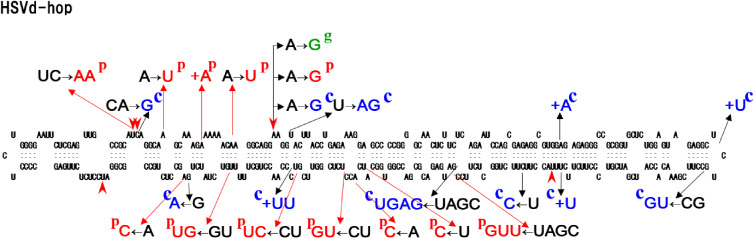
(Color online) Comparison of nucleotide variations of five HSVd variants on the predicted secondary structure. The changes and positions of the nucleotides in HSVd-g (green letter with g), HSVd-pl & -pe (red with p), and HSVd-c & cit (blue with c) are plotted on the predicted secondary structure of HSVd-h (black).

In search of the possible reservoir plants for HSVd, 105 specimens from 25 families, including weeds or wild plants growing in severely infested hop yards were tested, but all except for cultivated hops were negative.^158)^ Meanwhile, a viroid resembling HSVd having a similar host range and showing symptoms in cucumber plants identical to those induced by HSVd was detected from cultivated grapevines introduced into Japan from France, West Germany, Austria, Hungary, China, and the U.S.A., as well as from those cultivated in Japan.^159–161)^ Of 32 grapevine samples examined, 28 (88%) were positive with quite a high infection rates and detected either from healthy and diseased plants, such as those showing symptoms of fleck, corky bark, or leafroll. The complete nucleotide sequence revealed that a single-stranded circular RNA from grapevines consists of 297 nucleotides, the same size as HSVd type species from hops (HSVd-h), with a single-nucleotide substitution at position 54 from A to G. Namely, it turned out to be a point mutant of HSVd-h and was named HSVd-grapevine (HSVd-g).^161)^ Furthermore, a similar RNA was also found to be infecting citrus, plum, and peach trees cultivated in Japan. Analysis of the complete nucleotide sequence revealed that they were all HSVd variants and were named HSVd-citrus (HSVd-cit),^162,163)^ HSVd-plum (HSVd-pl),^164,165)^ and HSVd-peach (HSVd-pe).^165)^ HSVd-cit consisted of 302 nucleotides; HSVd-pl and HSVd-pe consisted of 297 nucleotides, which differed from HSVd-h at 17 and 19 positions, respectively (Fig. 5). As in the case of HSVd-g in grapevine, HSVd-cit-infected citrus trees were asymptomatic but had a quite high infection rate. In contrast, HSVd-pl exhibited fruit disorders called dapple fruit in Japanese plum cvs. “Taiyo” and “Ohishiwase-sumomo”, yellow flesh disorder of fruit in “Soldam”, and dapple fruit disease in Japanese peach cv. “Asama-hakutou”.^166)^

**Figure 6.**
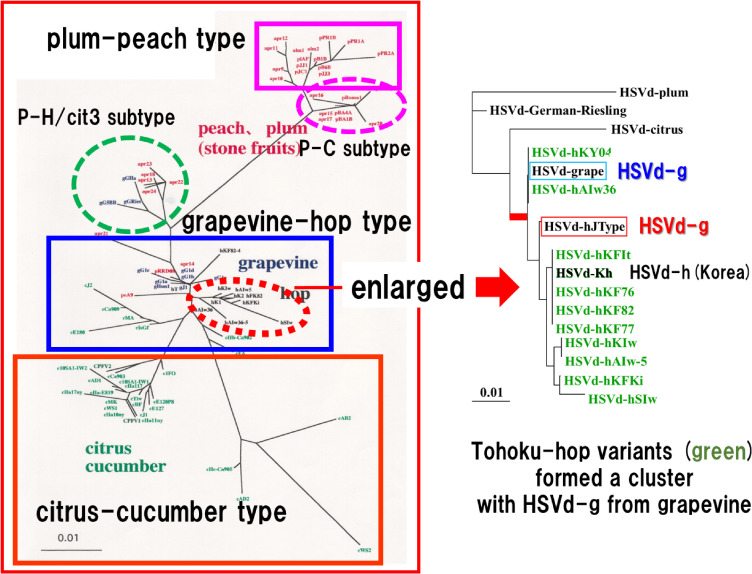
(Color online) A molecular phylogenetic tree of HSVd variants. HSVd variants isolated from hop, grapevine, citrus, plum, peach, cucumber, and others are divided into three major types; *i.e.*, grapevine–hop (blue rectangle), citrus–cucumber (red rectangle), and plum–peach (pink rectangle) types. Two sub-types, plum–hop (green circle), and plum–citrus (pink circle), thought to have arisen by recombination, are also proposed. The red dotted circle indicates the position of the natural variants isolated from the hops in the Tohoku region. The details of a cluster, including Tohoku-hop variants (green letters), are enlarged to the right panel.

These results indicated that HSVd infects fruit trees such as grapevine, citrus, plum, and peach widely. These infections were most often asymptomatic but caused serious fruit disorders on some sensitive cultivars of plum and peach. Since the discovery of PSTVd, seven species of viroids had been reported around the world at that time, but many of them were considered to be highly localized pathogens because their known geographic distribution was limited to small areas. HSVd was also considered to be a pathogen peculiar to Japan because no outbreak had been recorded in European countries or the United States, which have a long history of hop cultivation. However, the series of studies described above have completely changed the concept and revealed that HSVd is an important pathogen widely distributed around the world, infecting cultivated grapevines and citrus trees asymptomatically while causing serious disease problems in hops, plums, and peaches. In addition, these studies revealed that the nucleotide sequence of HSVd variants shows host specificity, and they were proposed to be divided into three major types (*i.e.*, grapevine–hop type, citrus–cucumber type, and plum–peach type) (Fig. 6).^165,167)^ Since then, HSVd has been isolated from 18 species of plants in 37 countries around the world, which include one vegetable plant, one perennial herbaceous plant, one nut tree, one flowering tree, and 14 fruit trees. To date, all HSVd mutants reported worldwide belong to one of the three types, but it has been suggested that among the variants isolated from stone fruits in China and European countries, there are at least two recombinant types that are believed to have arisen between the major three types.^168,169)^ HSVd is now known to have the widest host range among all viroid species. It is adaptable to the diverse host environments and has come to be recognized as a viroid that causes serious diseases, especially in fruit trees.^170)^

#### Grapevine origin of hop stunt disease—Host adaptation and molecular evolution of hop stunt viroid.

In the surveillance of HSVd epidemiology conducted in the late 1990s in the *Tohoku* district of Japan, where HSD was endemic, nine isolates of HSVd, five from Fukushima and four from Iwate were selected for sequencing, and a total 46 complete cDNA sequences were determined.^171)^ The nucleotide sequence of these HSVd variants appeared to be diverse. The HSVd population in hops in the region formed a quasi-species consisting of six predominant variants and a variety of minor variants. Each variant consisted of 296–301 nucleotides and were novel and differed from the reference variant of HSVd-h by 1–7 nucleotides. A molecular phylogenetic analysis including 44 HSVd sequences from various hosts registered in the DNA database at that time revealed that all the variants newly detected from hops in the *Tohoku* district were most closely related to the variant from grapevine, HSVd-g (Fig. 6). The results strongly suggested that the viroid latently infecting cultivated grapes may have been a source of HSD transmission in Japan.^171)^

**Figure 7.**
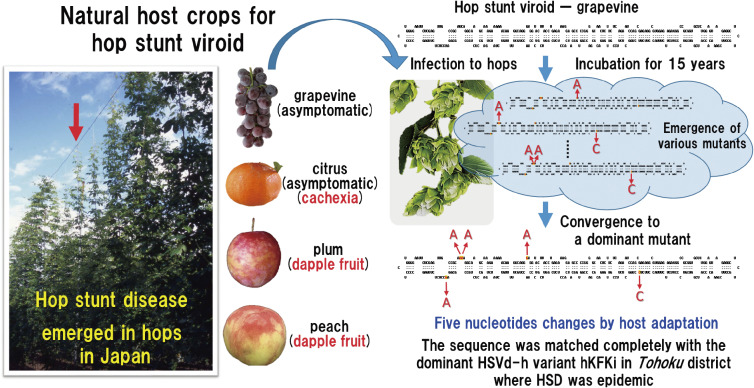
Schematic representations of HSVd variants in natural hosts and identification of a possible origin for HSD epidemic in Japan.

Four major HSVd variants from hop, grapevine, citrus, and plum (*i.e.*, HSVd-h, HSVd-g, HSVd-cit, HSVd-pl) were inoculated into virus- and viroid-free hop plants (cv. “Kirin II”) to assess the potential risk of HSVd variants infecting fruit trees to hop host. Infected hop plants were cultivated in an experimental yard over the next 15 years (a period from 1993 to 2007), and changes in the growth of infected hop vines and α-acid content in cones were analyzed every year. Plants infected with HSVd-h and HSVd-g both showed similar levels of mild stunting and leaf curling. Those with HSVd-cit showed the severest symptoms, and HSVd-pl was in the middle. Namely, the effect on plant growth was apparently different for each variant. In contrast, the α-acid content of all infected hop plants decreased to 46–55% of that of healthy from the second growing season and then remained low in the range of 50–60% of the healthy plants. These results indicated that all HSVd variants that asymptomatically infect fruit trees are at risk of causing HSD in hops.^172)^ Meanwhile, analysis of the nucleotide sequences of HSVd progeny recovered from infected hop plants every year revealed that new sequence variants began to appear 5 years after infection, and multiple HSVd sequence variants co-existed in infected hop plants. Furthermore, with the passage of years, the transition phenomenon of the dominant variants, in which the dominant variant in the population changed to a new type, was also observed (Fig. 7). In particular, the dominant variant in hop plants inoculated with HSVd-g changed one after another in various combinations of the nucleotide at positions 25, 26, 54, 193, and 281. During the 15 years of the observation period, 10 different sequence variants emerged and finally converged to one predominant variant in which all the five nucleotides were changed.^172)^ In HSVd-h, as in HSVd-g, the four nucleotides at 25, 26, 193, and 281 changed one after another during the 15 years of persistent infection and finally converged to the same dominant variant as HSVd-g. That is, both HSVd-g and HSVd-h (single-nucleotide variant of HSVd-g at position 54) changed to the same variant during hop infection. In this experiment, however, a natural isolate of HSVd-g was used for infection, so a question had arisen whether the detected mutants were newly created by adaptation or enriched from existing mutants present in the natural HSVd-g population. Therefore, the experiment was conducted using an *in vitro* transcript of HSVd-g as an inoculum. Again multiple hop plants infected with HSVd-g were planted in a yard and maintained for 10 years. During the observation period, 66 mutation positions were identified, including five major mutation hotspots at positions 25, 26, 54, 193, and 281, and all the mutations identified were present in the natural HSVd-g propagated in hops.^172)^ Ten different sequence variants that emerged in experiments with native HSVd-g mutants were also detected in this experiment, indicating that the natural HSVd-h variants found in commercial hops are *de novo* mutants generated by the same process through which HSVd-g adapted to a hop host and not mutants selected from minor variants pre-existing in the original inoculum. Of note, the nucleotide sequence of the dominant variant finally emerged after adaptation was matched perfectly with one of the dominant natural variant (hKFKi) isolated from hop yards in the *Tohoku* district in Japan and also with those reported from hops in the United States and China, where HSD became epidemic from the early 21st century.^7,151,172,173)^ On the other hand, HSVd-pl and HSVd-cit also changed nucleotides at positions 5 and 11, respectively, one after another during 15 years of the observation period. Still, both changed to dominant mutants different from HSVd-g and HSVd-h.^172)^

In search of the origin of the causal agent for HSD, HSVd was further surveyed in wild hops (*H*. *lupulus* var. *cordifolius*) and crimson glory vine (*V*. *coignetiae*) native to Japan, but those collected from several places in Japan were all negative for HSVd. In addition, among the old cultivated grapevines in Japan, one of the oldest vine trees of cultivars “Koshu” and “Zenkoji” grown on their own roots were also found to be HSVd negative.^174)^ These facts suggested that HSVd did not originally exist in grapevines grown wild or cultivated in Japan but was thought to have been brought into the country from abroad in recent years (no later than 150 years) along with cultivated grapevines asymptomatically infected with HSVd introduced for commercial or breeding purposes.

In summary, the result of molecular phylogenetic analysis of HSVd variants from hops and grapevine, in addition to circumstantial evidence that Japan’s major hop-producing areas overlap with grapevine-producing areas and they are often cultivated in close proximity, suggested that HSVd-infected grapevines were most likely a reservoir of the HSD epidemic in Japan (Fig. 6).^171)^ Analysis of the transition of host adaptive mutations that HSVd-g, HSVd-h, HSVd-cit, and HSVd-pl produced during persistent infection in hops further confirmed that HSVd-g asymptomatically infecting cultivated grapevines may be the origin of the hop stunt epidemics in Japan. Because the hop-adaptive mutant of HSVd-g that emerged *de novo* in the experiment completely matched the variant that dominated in hop yards in HSD endemic areas in Japan, as well as those in the United States and China, it was suggested that there may be some inevitability, but not by chance, in the molecular process by which HSVd-g adapts to the cellular environment of hop host.

In 2004, an HSD epidemic was first recognized in hop yards in Washington State in the United States,^151,152)^ and another outbreak was also confirmed in Ohio State in 2017.^175)^ HSD epidemics have also been reported in other major hop growing areas worldwide such as Xinjiang in China^173)^ and Slovenia in Europe.^174)^ In case of the Slovenian HSD epidemic first recognized in 2007, HSVd-cit type variant was detected from HSD-symptomatic hops, and subsequent follow-up detected a variant of CBCVd, both known as citrus-infecting viroids. Because the primary outbreak of the new disease occurred in a hop yard established on the site of a former waste dump (Radišek, 2015, Express Pest Risk Analysis: Hop stunt viroid (HSVd) on hop (*Humulus lupulus*). https://www.gov.si/assets/organi-v-sestavi/UVHVVR/Zdravje-rastlin/Organiziranost-zdravstvenega-varstva-rastlin/Ocena-tveganja/Sprejete-ocene-tveganja/Ocena_tveganja_-HSVd.pdf), it has been suggested that the remains of imported citrus fruits or plants may have been a source of transmission.^176,177)^ These HSD epidemics represent a growing threat to hop cultivation globally (Parkinson & Reed, 2013, Rapid pest risk analysis for *Hop stunt viroid*. https://secure.fera.defra.gov.uk/phiw/riskRegister/downloadExternalPra.cfm?id=3881; O’Neal, 2015, Pest management strategic plan for U.S. hops. https://www.usahops.org/img/blog_pdf/320.pdf).^178)^ HSVd variants themselves are pervasive and have spread worldwide as reported so far^170)^; therefore, any incident in which a viroid infecting grapevines or citrus spreads across species barrier and causes a new disease such as “hop stunt” or even another is at risk of emergence anywhere in the world.

### 3.2 Diversity of potato spindle tuber viroids in a wide range of natural host plants.

In addition to the nucleotide sequences or molecular motifs reported so far to be associated with the molecular functions of viroids, various mutations in the nucleotide sequence have also been reported widely around the world. Although the molecular functions of these variations remains unclear except for some limited cases, they seem to involve important information on the molecular processes of differentiation, evolution, and adaptation of the viroid genomic RNA in the host environment. This section focuses on the nucleotide sequence variations of PSTVd and discusses their association with pathogenicity.

In the early 1990s, a new variant of PSTVd (N) was detected in the Netherlands from healthy-looking pepino plants (*Solanum muricatum*) grown from seeds imported from overseas.^179)^ The variant consisted of 356 nucleotides; *i.e.*, three nucleotides shorter than normal strains, and it differed from the KF6 variant with three insertions, six deletions, and 14 nucleotide exchanges. Sequence analysis of five new field isolates of PSTVd revealed that the chain length varied between 356 and 360 nucleotides.^180)^ A Polish collection of PSTVd greenhouse isolates with severe, intermediate, and mild phenotypes consisted of multiple sequence variants; *i.e.*, s23, s27, i4 and i2 from severe, i2, i3 and i4 from intermediate, and M from mild, in which the variants s23 and s27 were shown to produce severe symptoms, i2, i3, and i4 produced intermediate symptoms, and M produced mild symptoms.^181)^

Then, early in the 21st century, PSTVd was detected from tomato and capsicum in New Zealand, and both isolates shared 100% nucleotide sequence identity, suggesting that they had the same origin, probably from infested seeds.^182,183)^ Their sequences were 1.7% different from a tomato isolate from the Netherlands and 3.7% different from a *Solanum* isolate from Australia.^182,183)^ In addition, since 2006, PSTVd variants have been discovered one after another from wider a range of hosts other than potatoes across the world, especially in European countries; *i.e.*, tomato and various ornamental plants belonging to *Solanaceae* and *Asteraceae* in Europe, Turkey, U.S.A., Canada, India, Japan, and so on.^62,184–187)^ Furthermore, large-scale surveys of PSTVd infection in potato conducted in Russia, China, and Turkey identified 17 different sequence variants in Russia and 42 different sequence variants including 30 novel ones in China, and comprehensive phylogenetic analysis uncovered a close relationship between the Chinese variants and those isolated from Russia.^188–190)^ Further analysis of the nucleotide sequence of more than 100 Russian PSTVd isolates represented 42 individual sequence variants, each containing 1–10 mutations with respect to the intermediate strain. Twenty-one isolates contained a mutation found only in Russian and Ukrainian isolates; *i.e.*, A to C substitution at position 121 (A121C). Many of them also contained deletion of one of the three A residues occupying positions 118–120 plus replacement of A at position 121 with either U or C (A120–, A121U/C). These mutations were phenotypically neutral, *i.e.*, symptoms expression in “Rutgers” tomato were unaffected.^191)^

**Figure 8.**
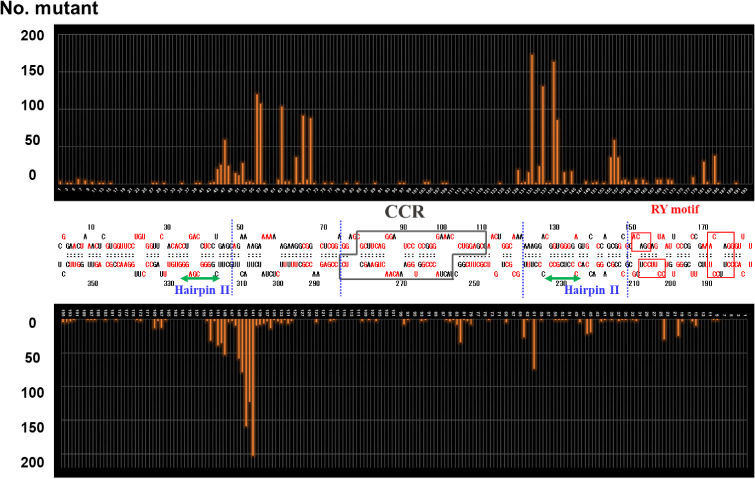
(Color online) Variations in natural PSTVd populations and their frequency. Among 309 complete nucleotide sequences deposited in the database, 210 nucleotide positions (red) were conserved, but 149 nucleotide positions were variable. The vertical axis shows the number of mutants that have mutations in that position.

**Figure 9.**
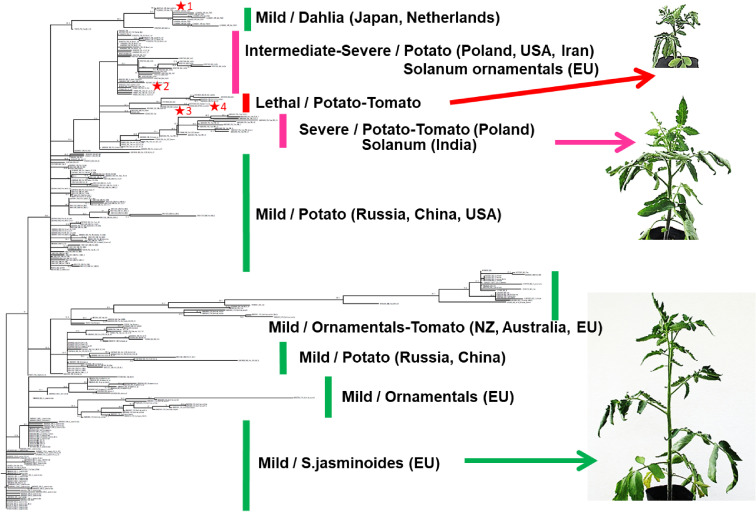
(Color online) A maximum likelihood phylogenetic trees of 309 complete PSTVd variants registered in the database. The pathogenicity, host, and country of the representative variants in each cluster are shown; *i.e.*, green, pink, and red bars represent mild, intermediate - severe, and lethal pathogenic to tomato, respectively. Red stars 1, 2, 3, and 4 represent the location of Dahlia (mild), DI (Diener’s isolate; Intermediate), RG1 (lethal), and KF440-2 (lethal), respectively.

More than 300 sequences of PSTVd variants have now been deposited in DNA databases. Among these, more than 200 nucleotide positions were conserved, but the rest were variable. The rate of change varied depending on the position. It was generally high in the P and the V domains, with up to 60% or more variants mutated in some locations (Fig. 8). Phylogenetic analysis of more than 300 sequences of various PSTVd variants deposited in DNA databases revealed that those isolated since the 2000s from *Solanaceae* and *Asteraceae* ornamentals and those from potato analyzed recently in Russia and China formed a cluster with variants showing mild-type symptoms on tomato. On the other hand, although bootstrap support was not high, variants showing severe type symptoms on tomato and those showing lethal-type symptoms formed clusters, respectively (Fig. 9).

In summary, just as the diverse and widespread subclinical hosts of HSVd existed as sources of infection behind the HSD epidemics, it has now become clear that PSTVd also has a variety of subclinical hosts in *Solanaceae* and *Asteraceae* ornamentals. In addition, various mild-type PSTVd variants were found to be widespread in potato growing areas around the world. Genome- or nucleotide sequence-based typing of pathogenicity of PSTVd variants has become an important issue for risk assessment.

### 3.3 Host factors affecting viroid–host adaptation.

Regarding biotic stresses, *i.e.*, host-specific processing of viroid sequences or viroid–host adaptations, some notable reports have already been published. Analysis on PSTVd replication in cultured tobacco cells demonstrated that substitutions of U257A and C259U locating in the lower strand of the CCR each enhanced PSTVd replication by 5- to 10-fold. A U257C substitution also led to enhanced replication in tobacco cells, but neither of U257G, C259A, nor C259G substitutions did. Of note, all of the nucleotide changes did not alter PSTVd replication levels in *N. benthamiana* cells. These results provided insights about PSTVd structures in the CCR that modulate replication efficiency in adapting to a specific host.^192)^ Prolonged infection of CEVd in tomato hybrid (*S. lycopersicum* × *S. peruvianum*) resulted in the production of two new enlarged variants in which a locus near the TR region was duplicated. These elongated variants can replicate in a tomato hybrid, but they failed to transmit to citrus hosts of the genera *Citrus*, *Poncirus* or *Fortunella*, suggesting host-directed processing of CEVd.^193)^ Analysis of the genetic diversity of CEVd populations infecting various citrus hosts, trifoliate orange, and sour orange after 10 years of prolonged incubation revealed that the amount and composition of genetic diversity generated after 10 years of evolution varied between these two hosts and was markedly different from the characteristics of the original Etrog citron population. Meanwhile, when the populations isolated from trifoliate orange and sour orange trees were back-inoculated into Etrog citron, the genetic diversity reverted to what is basically indistinguishable from the diversity characteristics of the original Etrog citron in a short period of time. Consequently, these results supported the notion that the composition and structure of the viroid population is determined by the host in which they replicate.^194)^ Transmission of a natural AFCVd isolate from hop to tomato, cucumber and wild hop resulted in the production of host-specific sequence changes in their population structures. The major variants in tomato and cucumber were almost identical, and the one in wild hop was very similar to the one in cultivated hop. Detailed analyses of host-dependent sequence changes indicated that the major AFCVd variant in tomato emerged by selection of a minor variant present in the inoculum followed by one to two host-dependent *de novo* mutations. Comparison of the secondary structures of major variants in hop, tomato, and persimmon after transfer to tomato revealed that stem–loop structures in the left-hand half of the molecule were conserved, suggesting that maintenance of the structure is critical for infection to tomato host.^90)^ Infection with an infectious cDNA clone of Chaipayon-1 isolate of columnea latent viroid (CLVd) into different *Solanaceae* host plants resulted in the production of distinct quasi-species populations containing 19 major variants with an average relative abundance greater than 1%. Secondary structure prediction clustered all major variants into a tomato group with four loops (I, II, IV, and V), and a chili pepper group with four loops (I, III, IV, and V) in the TR domain, which was in contrast to the original Chaipayon-1 variant consisting five loops (I, II, III, IV, and V), suggesting that a host-specific mutation has occurred in the secondary structure of the TR domain in CLVd.^195)^

What will be the driving force in host environments that direct adaptive mutations or Darwinian selection in viroids? A hypothesis has been put forward that RNA silencing is an important selection pressure that shapes the evolution of viroid secondary structure.^97)^ In this hypothesis, RNA silencing in plants can play a central role in the evolution of viroid secondary structure. Namely, viruses achieve these functions by means of an array of coding proteins that suppress host defense represented by RNA silencing, whereas viroids–a non-protein coding RNA–ensure their evolutionary survival using an exclusively sequence and structure-based strategy. The following describe the possible involvement of RNA silencing in viroid–host adaptation.

Shifts in viroid population dynamics due to mutations over the course of infection were analyzed using PSTVd-RG1-infected tomato plants by high-throughput sequencing.^196)^ The 10 most abundant sequence variants expressed at different time intervals were identified. Some of them may have a positive effect on viroid accumulation by inducing RNA silencing of host defense-related genes. Other variants with mutations that can adversely affect the abundance of viroids have also been identified. The latter was hypothesized to be because the vd-sRNA derived from these variants can no longer target any host mRNA or change the target sequence from a host defense gene to another insignificant host gene.

As presented in the previous section, accidental transmission of HSVd from cultivated grapevine to hop caused host adaptation in which a variant of HSVd-g underwent convergent evolution in hop and produced a predominant hop-adapted variant HSVd-hKFKi containing five mutations at the positions 25, 26, 54, 193, and 281. Detailed analysis of the pathogenicity of HSVd-g and HSVd-hKFKi in their hosts and high-throughput sequencing of HSVd-specific small RNA (HSVd-sRNA) revealed that the five hop-adaptive mutations affect the pathogenicity of HSVd and also the biogenesis of HSVd-sRNA containing these mutations differently in cucumber and hop hosts.^111)^ According to the “survival of the fittest” paradigm of Darwinian evolution, in which natural selection favors the best-adapted replicators, it is predicted that HSVd-hKFKi should have a higher fitness than HSVd-g in hop. Results of the competition assays, however, only partially matched the predictions. Namely, contrary to the expectations, HSVd-hKFKi showed only marginal advantages over HSVd-g in its ability to replicate/accumulate in hop, the adaptive host; *i.e.*, both variants co-existed almost equally without out-competing the others. In addition, HSVd-hKFKi showed a reduced ability to replicate/accumulate in cucumber, an experimental host, and lost the ability to replicate stably in grapevine, an original host before adaptation to hop. Therefore, the five adaptive mutations that emerged in hop seemed to have an adverse effect upon the ability to replicate/accumulate in a single cell or move from cell to cell in other hosts. This phenomenon may represent a “trade-off” that occurs during the prolonged infection/adaptation process in hop, a new host environment for the variant. It was proposed that the finding that fitness of HSVd-hKFKi is similar to that of the original variant, HSVd-g, in hop may be explained by the evolution of viroid populations as quasi-species. Namely, host selection should result in a higher fitness population, not a higher fitness single sequence variant; *e.g.*, most host-selected single-nucleotide mutations in the population are expected to be deleterious or neutral, and only a few are beneficial.

Further analysis on the effects of the five adaptive mutations on the biogenesis of HSVd-sRNA revealed that the distribution of HSVd-sRNAs generated from genomic and antigenomic strand of HSVd-g and HSVd-hKFKi in hop and cucumber hosts was strongly biased toward several positions, particularly to those containing the five hop-adaptive mutations at positions 25, 26, 54, 193, and 281.^111)^ For example, in the antigenomic strand, compared with HSVd-g, HSVd-hKFKi produced less abundant HSVd-sRNAs containing the nucleotides 25/26, 54, or 281, both in hop and cucumber hosts. The results showed that hop-adaptive mutations of HSVd affected the biogenesis of HSVd-sRNA, and suggested that host adaptive mutations may have the effect of mitigating the degradation of antigenomic strand of HSVd by RNA silencing.

Not only biotic stresses but abiotic stresses such as heat also play a significant role in viroid–host adaptation. Heat treatment of hop led to HLVd degradation and, simultaneously, to a significant increase in sequence variations. Sixty-nine percent of mutations were localized in the left half and 31% in the right half of the secondary structure proposed for this viroid. A “hot spot” region was identified in a domain known as the P domain. Most mutations were predicted to destabilize HLVd secondary structure.^197)^ Thermal stress of PSTVd-infected *N. benthamiana* also led to the appearance of a broad range of PSTVd sequence variants, most of which accumulated in the left half of the molecule including the P domain.^198)^ That is, abiotic stress such as heat is thought to lead to destabilization of normal molecular processes in the cell that maintain optimally adapted viroid populations in normal growth conditions, resulting in the production of significant mutations in viroids.

## Postscript—Future prospects

At the time of their discovery, a general image of viroids was of something like a virus that had lost its outer capsid. Fifty years later, the unique molecular structure of single-stranded circular RNA has been elucidated, and various nucleotide sequences and structural motifs in the molecule have also been identified. Now, the general concept of the functional noncoding RNA nature of viroids has been well established as described in this review. However, understanding of their life phenomena appears as an ongoing effort; *i.e.*, elucidation of this novel RNA pathogen raises broader and more comprehensive questions. In fact, there are still some interesting issues that need to be answered in full; *e.g.*, the transcription start site for replication, the enzyme(s) for processing and ligation of the unit-length (+)-strands of pospiviroid from the replicative intermediate, or the mechanism by which avsunviroid, which replicates in chloroplasts, produces small RNAs. Furthermore, various nucleotide sequences and structural motifs involved in the functions of viroid RNA were identified, and a large number of host genes that are differentially expressed following viroid infection have been clarified, but the mechanisms by which viroids cause diseases remain largely unknown. Namely, as a basic point, various host factors that interact with multiple domains or motifs within the viroid RNA that underlie the expression of various functions of viroids such as autonomous replication, pathogenicity, and host adaptation await further elucidation. Research on these viroid properties and host interactions are expected to be applied to new technologies for controlling viroid diseases, such as the development of viroid-resistant plants.

Viroids were initially thought to be a somewhat specific pathogen localized in specific crops in limited areas, but it was demonstrated that there are various alternative host crops that harbor viroid(s) asymptomatically beyond these. Viroids are already highly adapted to a variety of host environments and survive by maintaining a semi-mutualistic relationship. On the other hand, they emerge as new diseases by accidental transmission to highly susceptible host crop(s) beyond the species barrier. Viroids by strict definition are currently known only in higher plants. So far, the discovery of new viroids has been conducted by combining specific gel electrophoresis techniques that can detect unique physicochemical properties of viroid RNAs, *i.e.*, a single-stranded circular RNA, and bioassays to confirm their autonomous replication to fulfill Koch’s postulate. In recent years, however, thanks to the development of next-generation sequencing technology and an algorithm that predicts the presence of novel single-stranded circular RNA *in silico*,^199)^ it has become possible to detect new or known viroids at low levels that was not achievable using conventional techniques.^200–203)^ In fact, it is interesting to note that some of them found by using such new technologies present in very low amounts in the infected plants. This may suggest that they are still at a primitive stage in terms of fitness. It would be of value to discover varieties of new viroids that go beyond the conventional concept and in a wider ranges of species. This is because it may provide information that arouses intellectual interests about the origin of viroids or the origin of life, given that viroids are considered to be a living fossil from the pre-cellular RNA world.

In conclusion, it has been clarified that viroids invade host cell and replicate/accumulate in the nucleus or organelle to produce various nucleotide variations in the process of adapting to new intracellular environments in novel hosts. Then, they are thought to exert a different levels of pathogenicity by interacting with host factors in various ways. Although as small as only 246–434 nucleotides, the molecular mechanism of viroid pathogenicity has turned out to be more complicated than previously imagined. Further understanding of the specific interactions of viroid RNAs with diverse and complex host gene expression networks is definitely essential. Meanwhile, looking at research fields other than viroids, the concept of functional non-coding RNA has also become more widely recognized in the world of biology, suggesting structural similarities between viroids and human miRNAs and possible involvement of single-stranded circular RNA structurally similar to viroid in human diseases, for example.^204–206)^ By incorporating advances in various research fields, viroids will continue to serve as an excellent model for studying how RNA structural elements and motifs express their functions and affect normal biological processes in the cell. Elucidation of the fundamental mechanisms underlying the pathogenesis and host adaptation of viroids is not only an urgent task for developing control strategies against viroids, for which there is still no effective control measure other than diagnosis, but also it has broad significance in the study of human health.
